# Frequency-Selective Fat Suppression Radiofrequency Pulse Train to Remove Olefinic Fats

**DOI:** 10.1007/s00723-013-0474-6

**Published:** 2013-07-19

**Authors:** Takayuki Abe

**Affiliations:** Molecular Imaging Center, National Institute of Radiological Sciences, 4-9-1 Anagawa, Inage-ku, Chiba, 263-8555 Japan

## Abstract

CHESS pulse can suppress the signal originating from aliphatic fat protons but cannot suppress the signal from olefinic fat protons, which is near the resonance frequency of water protons. Adipose tissue contains various fat species; aliphatic fat comprises about 90 % and olefinic fat about 10 % of adipose tissue. Thus, CHESS pulse cannot be used to suppress the signal from adipose tissue completely. The purpose of this study was to find a method to suppress the signal from adipose tissue completely. The Fatsat train pulse, created with an arbitrary flip angle and insensitive to B1 inhomogeneity, was used. Because B1 inhomogeneity is larger on higher field magnetic resonance imaging, the fat suppression radiofrequency pulse needs to be B1-insensitive. To investigate a percentage of olefinic fat in adipose tissues, the excitation frequency of the Fatsat train pulse was varied from −240 to +400 Hz and the images and fat-suppressed images were obtained. The presence of olefinic fat comprising about 10 % of abdominal adipose tissue was identified. The result agreed with some previous papers. Complete fat suppression could be achieved by partial (10 %) inversion of longitudinal aliphatic fat magnetization and by canceling out the two fat magnetizations. The flip angle was identified to about 95°. In conclusion, the cause that the signal from adipose tissues cannot be suppressed completely has been found. Improved images that signals from adipose tissues were suppressed completely have been demonstrated. This technique can also be applied to several pulse sequences.

## Introduction

Fat suppression is used in routine clinical magnetic resonance imaging (MRI). Adipose tissue appears bright in T_1_- and T_2_-weighted imaging, and a chemical shift artifact occurs in diffusion-weighted imaging (DWI). These signals can interfere with the diagnosis of certain diseases. Thus, a method is needed to suppress completely the signal from adipose tissue in these applications. A chemical shift-selective (CHESS) radiofrequency (RF) pulse is commonly used for fat saturation (Fatsat) [1, 2]. To suppress the signal from lipid (aliphatic fat), which precesses at a lower frequency of 3.5 ppm than water, the CHESS pulse nutates the lipid magnetization to the transverse plane, while leaving the water magnetization unperturbed along the longitudinal axis. A spoiler gradient pulse is subsequently applied to diphase the excited lipid signal. Adipose tissue has a complex chemical spectrum that contains a number of different spectral components. Generally, there are two components of fatty tissues, lipid (aliphatic fat) and olefinic fat, which precesses at the same frequency as water [3, 4]. Although the CHESS RF pulse can suppress the signal from aliphatic fat, it cannot suppress the signal from olefinic fat. Therefore, the conventional CHESS RF pulse does not completely suppress the signal from adipose tissue, and a residual signal from olefinic fat remains.

**Fig. 1:**
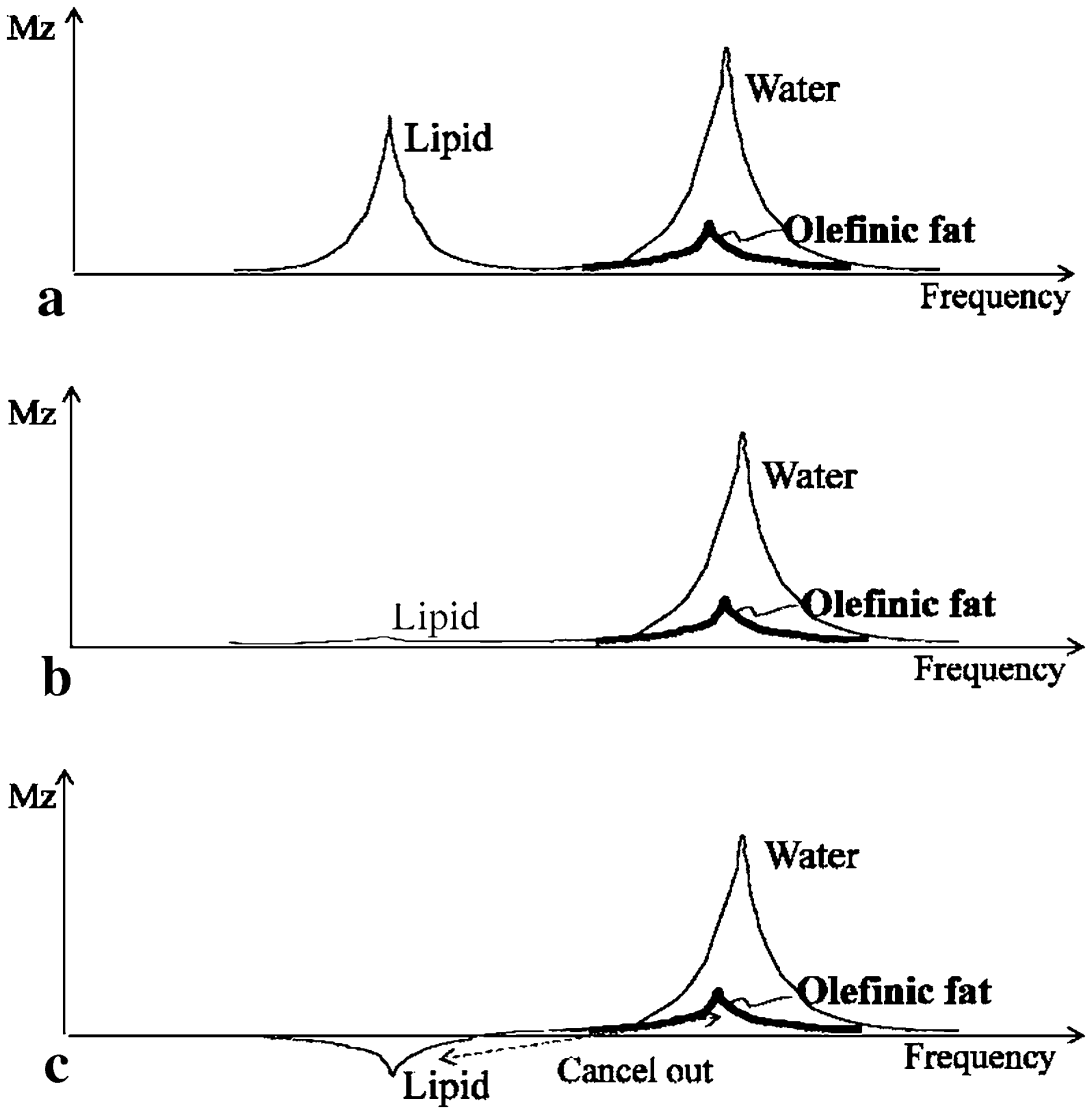
Diagram of the relationships between lipid, water, and olefinic fat (**a**). Spectrum in using a conventional CHESS RF pulse (90°) is shown (**b**). The spectrum shows that the longitudinal fat magnetization is partially inverted to suppress completely the signal from adipose tissues (**c**)

The purpose of this study was to investigate the influence of olefinic fat in adipose tissue using a CHESS RF pulse and to identify a method to suppress completely the signal from adipose tissue. The frequency-selective B_1_-insensitive Fatsat train pulse by creating an arbitrary flip angle (FA) was used to investigate the presence ratio of olefinic fat by varying the offset frequencies [5, 6]. The presence of about 10 % olefinic fat in adipose tissue was demonstrated. This result agrees with values reported previously. This study also found that the inversion of longitudinal aliphatic fat magnetization by 10 % could cancel out the olefinic fat magnetization by 10 %, as shown in Fig. 1, and that complete fat suppression could be realized. Images of the liver and breasts with complete fat suppression were obtained. The results may also be applicable to the conventional CHESS RF pulse.

## Methods

**Fig. 2:**
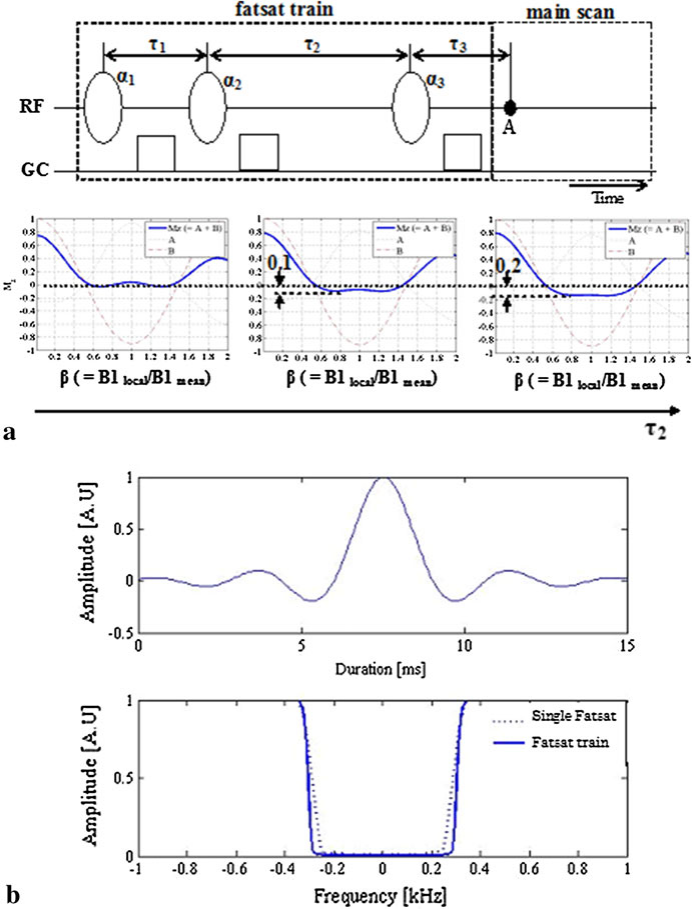
Diagram of the Fatsat train comprising three spectrally selective RF pulses with different FAs (**a**). Spoiler pulses are used to destroy any transverse magnetization between each RF pulse. An excitation RF pulse of the main scan is commonly executed at time A. The longitudinal fat magnetization (*M*_z_) by varying *τ*_2_ is shown. The subpulse of the Fatsat train and the frequency profiles are shown (**b**)

A Fatsat train pulse, as shown in Fig. 2a, was used. The Fatsat train pulse was B_1_-insensitive and an arbitrary FA could be used by adjusting the time interval *τ*_2_, as shown in Fig. 2 [5, 6]. The time intervals *τ*_1_ and *τ*_3_ were 17 and 13 ms, respectively. The default *τ*_2_ was 17 ms, at which the net FA was produced at 90°. A sinc-shaped RF pulse in the Fatsat train was used. The sinc-shaped RF pulse designed had a ripple amplitude of 1 % and a time bandwidth product of nine. A 15-ms sinc-shaped RF pulse duration was selected (Fig. 2b). The RF bandwidth was about 600 Hz (Fig. 2b). The FA for three RF pulses was optimized using Eq. (1). The FA at α_1_, α_2_, and α_3_ was used at 117°, 79°, and 180°, respectively.

1 $$ \begin{aligned} M_{{{\text{z}},3}} &=  \, \left[ {\left( {1 \, - \, \exp (-t/{\text{T}}_{1} )} \right)\cos (\beta \cdot \alpha_{1} )\cos (\beta \cdot \alpha_{2} )\cos (\beta \cdot \alpha_{3} )} \right. \\ &\quad \left. { + \, \left( {1 \, - \, \exp (-\tau_{1} /{\text{T}}_{1} )} \right)\cos (\beta \cdot \alpha_{2} )\cos (\beta \cdot \alpha_{3} ) \, - \, \cos (\beta \cdot \alpha_{3} )} \right]\exp \left( {-(\tau_{2} + \tau_{3} )/{\text{T}}_{1} } \right) \\&\quad  - \, \left( {1 \, - \, \cos (\beta \cdot \alpha_{3} )} \right)\exp (-\tau_{3} /{\text{T}}_{1} ) \, + \, 1, \end{aligned} $$

where *τ*_1_ is the time interval between the first subpulse and the second subpulse, *τ*_2_ is the time interval between the second subpulse and the third subpulse, and *τ*_3_ is the time interval between the third subpulse and an excitation RF pulse of the main scan.

**Fig. 3:**
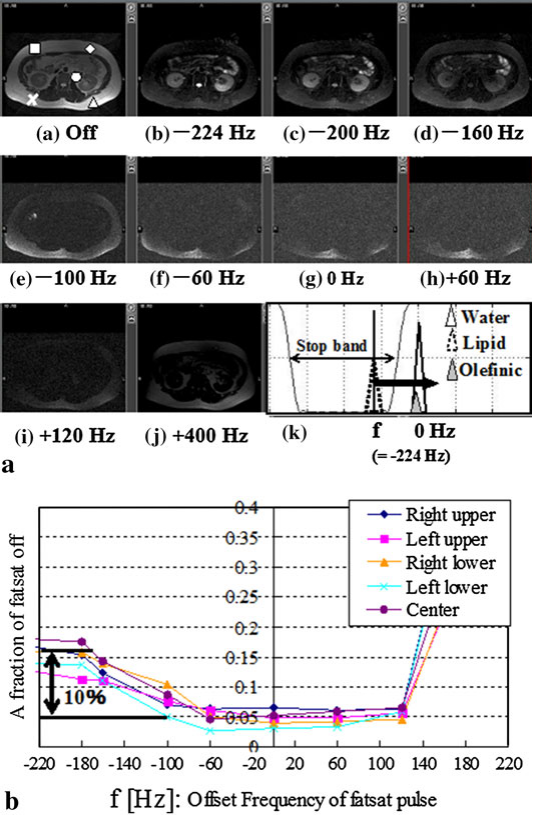
Abdominal fat-suppressed images obtained by varying the offset frequency from −224 to +400 Hz. The relationship between the offset frequency and the frequency response of the Fatsat train is shown (**a**(*k*)). The plot of the signal normalized by the signal without the CHESS pulse as a function of the offset frequency is shown (**b**)

The net FA was adjusted to 90°, and the time interval *τ*_2_ was 17 ms. To identify the reason why adipose tissues cannot be suppressed completely, the resonance frequencies were investigated in adipose tissue of health volunteers. To demonstrate the spatial differences between resonance frequencies in adipose tissue, the offset frequency (**f** in Fig. 3a(k)), which was the excitation frequency of the Fatsat train, was varied from −240 to +400 Hz, and the fat-suppressed images were acquired. To analyze the fraction of the signal from the remaining fat, the fraction of the image without the Fatsat pulse was measured and calculated for five areas in several adipose tissues, as shown in Fig. 3a (identified by the square, circle, triangle, cross, diamond). To reduce B_0_ inhomogeneity, an active shimming was performed and the same condition was maintained during the experiments. B_1_ inhomogeneity was not considered because the Fatsat train pulse used was B_1_-insensitive.

Fat-suppressed images using different *τ*_2_ values were obtained in studies of adipose tissue to identify the optimal FA that achieved complete fat suppression. Active shimming was performed and the same conditions were maintained in studies of adipose tissue in human volunteers.

The Fatsat train pulse with an optimized FA was applied to a 3D T_1_-weighted gradient echo sequence (TIGRE) and a 2D fast spin echo (FSE) sequence. MRI scans of the liver and breasts of healthy volunteers were conducted after permission was obtained with informed consent. The scan parameters were as follows: 3D TIGRE: *TR* = 4.7 ms, *TE* = 1.7 ms, FA = 12°, thickness = 6 mm, acquisition matrix = 224 × 224 × 32, SENSE factor = 1.8, and scan time = 23 s; 2D FSE: TR/TE/FA = 5,500 ms/93 ms/90°, thickness = 8 mm, number of slices = 18, acquisition matrix = 256 × 192, interecho time = 8 ms, echo train length = 30, and scan time = 23 s. A Fatsat pulse and the Fatsat train were used for the comparison. Imaging was performed on a 1.5-T MRI scanner (Echelon, Hitachi Medical Corporation). The RF transmitter coil was a quadrature volume coil, and the RF receiver coil was either an eight-element torso coil or a seven-element breast coil.

## Results

### Fraction of Remaining Adipose Tissues by Varying Offset Frequency

Figure 3a(b–j) shows abdominal images at varying offset frequencies of the Fatsat train. Figure 3a(f) shows the relationship between the offset frequency and the frequency profile. Figure 3b shows a plot of the fraction of remaining adipose tissues normalized by the signal without the Fatsat pulse as a function of the offset frequency for the five areas shown in Fig. 3a(a). The adipose tissue was suppressed almost completely at −224 Hz for the offset frequency, but the percentages of remaining adipose tissues for the five areas were 10–15 %. The percentages at −100 Hz of the offset frequency were 10 %. The signal from renal and other tissues was suppressed almost completely at −100 Hz of the offset frequency. The percentages at −60 Hz of the offset frequency were <5 %, and the signals from adipose tissue were suppressed completely. The bright areas in the subcutaneous tissues represent the distribution of the receiver coil sensitivity. The differences in the percentages between −220 and −60 Hz were about 10 %.

### Optimization of the FA of the Fatsat Pulse

**Fig. 4:**
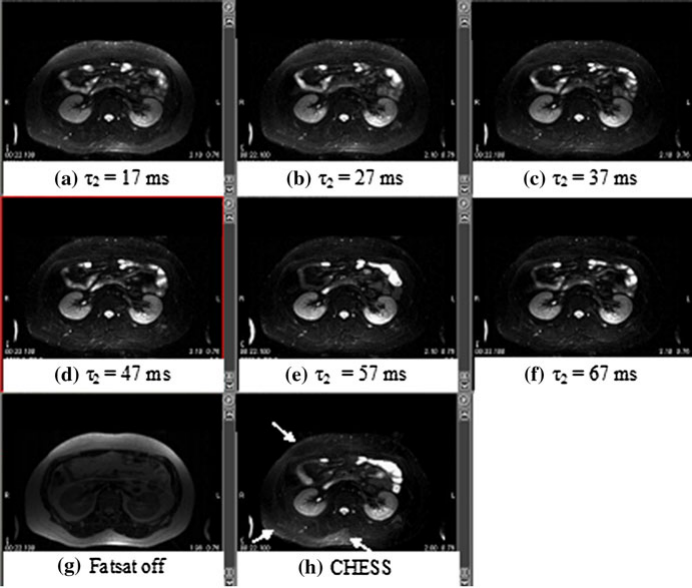
Fat-suppressed images in the abdomen of a volunteer using the Fatsat train with different *τ*_2_ (**a**–**f**). Images without Fatsat (**g**) and with a conventional CHESS RF pulse (**h**) are shown for comparison. Fat suppression with the Fatsat train is more uniform (**a**–**f**). On the other hand, the fat-suppressed image with a conventional CHESS RF pulse is nonuniform (*white arrow*). The signal from residual fatty tissues changed with *τ*_2_ and was null at *τ*_2_ = 47 ms (the net FA corresponds to 95°)

Figure 4 shows abdominal images at various FAs (the interval for *τ*_2_) of the Fatsat train. When *τ*_2_ was 17 ms (FA is identical to 90°), a few signals from adipose tissue in the subcutaneous tissues remained (Fig. 4a). When *τ*_2_ was 47 ms (FA is identical to 95°), the signals from adipose tissue were suppressed completely (Fig. 4d). When FA was 95°, the longitudinal fat magnetization was identical to −10 % M_0_. Images with the Fatsat train were spatially uniform. In contrast, images with the CHESS RF pulse were not uniform (arrow in Fig. 4h).

### Demonstration of Complete Fat Suppression in Human Volunteers

**Fig. 5:**
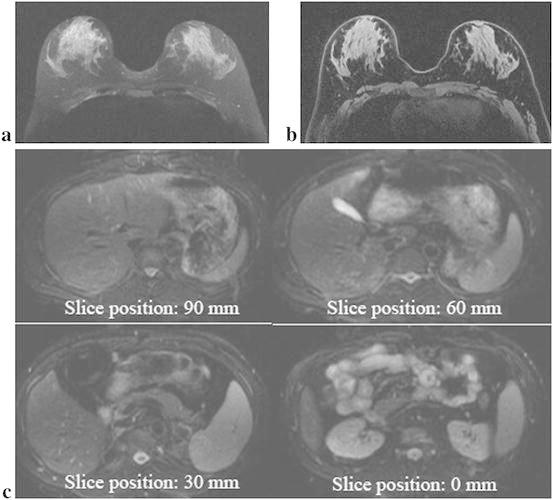
Fat-suppressed images of breast when the Fatsat train was applied to T_1_-weighted FSE (**a**) and T_2_-weighted FSE (**b**) sequences are shown. Fat-suppressed images of the abdomen obtained at several slice positions are shown (**c**). Complete fat suppression is demonstrated

Figure 5a, b shows breast images with the Fatsat train with the *τ*_2_ at 47 ms. The signals from adipose tissue were suppressed completely in both T_1_- and T_2_-weighted images. Figure 5c shows abdominal images scanned at different slice positions. The signals from adipose tissues were suppressed completely at several slice positions. Complete fat suppression was demonstrated.

## Discussion

The focus of this research was to identify a method to suppress completely the signal from olefinic fat in adipose tissue. To accomplish this objective, a spectrally selective fat suppression RF pulse was used to measure the percentage of olefinic fat in adipose tissue in human volunteers. This study produced two major findings.

First, the reason why the signal from adipose tissue cannot be suppressed completely by a CHESS RF pulse with an offset frequency of −224 Hz was identified. The cause is the presence of olefinic fat, which precesses near the water resonance frequency. Olefinic fat comprises about 10 % of adipose tissue. This result is consistent with previous reports [3, 4]. Although this paper shows the results from adipose tissue in only one volunteer, the results obtained from other volunteers were the same as those shown in Fig. 3.

Second, to suppress the signal from adipose tissue completely, the longitudinal fat magnetization was inverted to −0.1 M_0_ (10 %), which was identical to an FA of 95°. This was achieved using a B_1_-insensitive Fatsat train pulse and an arbitrary FA. It is found that the inversion of longitudinal aliphatic fat magnetization by 10 % could cancel out the olefinic fat magnetization by 10 %, as shown in Fig. 1, and that complete fat suppression was demonstrated in this study. In other words, we can suppress the signals from olefinic fat, which precesses at the same frequency as water if we use the Fatsat train pulse with an FA of 95°. The Fatsat train pulse can be applied to several pulse sequences. If the conventional CHESS RF pulse is used, the signal from fat will remain because of B_1_ inhomogeneity. In contrast, if an adiabatic inversion pulse is used, the inversion time (TI) must be adjusted [7–11], and TI can represent large amount of extra time. Improved Dixon method can separate the signal from several fats, respectively [12], but the reconstruction of the method is time-consuming compared to the frequency-selective fat suppression RF pulse.

The limitation of this study is that the fraction of the olefinic fat may vary between individuals. Future work should include more subjects with a range of fat levels to confirm whether this method can be applied to all people.

In conclusion, the reason why the signal from adipose tissues cannot be suppressed completely by CHESS RF pulse with an offset frequency of –224 Hz is the presence of olefinic fat, which comprises about 10 % of adipose tissue. To suppress the signal from adipose tissues completely, longitudinal fat magnetization should be inverted to −0.1 M_0_ (10 %), which is identical to an FA of 95°. This was realized using a Fatsat train pulse with B_1_ insensitivity and producing an arbitrary FA.
